# Coenzyme Q10 Prevents Senescence and Dysfunction Caused by Oxidative Stress in Vascular Endothelial Cells

**DOI:** 10.1155/2018/3181759

**Published:** 2018-07-08

**Authors:** Jia Huo, Zhe Xu, Kazunori Hosoe, Hiroshi Kubo, Hiroki Miyahara, Jian Dai, Masayuki Mori, Jinko Sawashita, Keiichi Higuchi

**Affiliations:** ^1^Department of Aging Biology, Institute of Pathogenesis and Disease Prevention, Shinshu University Graduate School of Medicine, Matsumoto 390-8621, Japan; ^2^The Third Hospital of Hebei Medical University, Shijiazhuang 050017, China; ^3^The First Hospital of Hebei Medical University, Shijiazhuang 050030, China; ^4^Supplemental Nutrition Division, Pharma & Supplemental Nutrition Solutions Vehicle, Kaneka Corporation, Osaka 530-8288, Japan; ^5^Department of Advanced Medicine for Heath Promotion, Institute for Biomedical Sciences, Interdisciplinary Cluster for Cutting Edge Research, Shinshu University, Matsumoto 390-8621, Japan; ^6^Department of Biological Sciences for Intractable Neurological Diseases, Institute for Biomedical Sciences, Interdisciplinary Cluster for Cutting Edge Research, Shinshu University, Matsumoto 390-8621, Japan

## Abstract

Oxidative damage in endothelial cells is proposed to play an important role in endothelial dysfunction and atherogenesis. We previously reported that the reduced form of coenzyme Q10 (CoQ_10_H_2_) effectively inhibits oxidative stress and decelerates senescence in senescence-accelerated mice. Here, we treated human umbilical vein endothelial cells (HUVECs) with H_2_O_2_ and investigated the protective effect of CoQ_10_H_2_ against senescence, oxidative damage, and reduction in cellular functions. We found that CoQ_10_H_2_ markedly reduced the number of senescence-associated *β*-galactosidase-positive cells and suppressed the expression of senescence-associated secretory phenotype-associated genes in H_2_O_2_-treated HUVECs. Furthermore, CoQ_10_H_2_ suppressed the generation of intracellular reactive oxygen species (ROS) but promoted NO production that was accompanied by increased eNOS expression. CoQ_10_H_2_ prevented apoptosis and reductions in mitochondrial function and reduced migration and tube formation activity of H_2_O_2_-treated cells. The present study indicated that CoQ_10_H_2_ protects endothelial cells against senescence by promoting mitochondrial function and thus could delay vascular aging.

## 1. Introduction

Cardiovascular diseases (CVD) continue to be a leading cause of death and disability worldwide [1]. This trend is particularly pronounced among aged populations in many countries. Since CVD mortality rates increase with patient age, the aging process is recognized to be the largest risk factor for the development of CVD [2], especially atherosclerosis [3].

The term senescence, a process encompassing age-related and irreversible reductions in physiological functions and increasing mortality rate, is often used to distinguish these processes from chronological aging. Senescence is complex and involves metabolic changes and destruction of molecular and cellular homeostasis that can eventually lead to organ failure and death [4]. Recent studies in humans and animal models suggest that vascular aging/senescence leads to impaired endothelial functions associated with elevated oxidative stress and a proinflammatory phenotype [5]. Senescent cells can activate various types of proinflammatory cytokines, chemokines, growth factors, and proteases, which together form the senescence-associated secretory phenotype (SASP) [6].

Hayflick and Moorhead first described cellular senescence in 1961 when they observed a limited ability of cells to replicate *in vitro*, which they referred to as replicative senescence [7]. At the cellular level, senescence is a complex pathophysiological process that includes various factors, such as accumulation of DNA damage [8], oxidative stress [9], and activation of signaling pathways involved in the general aging process. Several treatments were subsequently identified that promote cellular senescence and induce the phenotype known as stress-induced premature senescence (SIPS). Reactive oxygen species (ROS) are formed as natural products of metabolism and have important roles in several cellular signaling and metabolic pathways, including mitochondrial oxidative phosphorylation, cell proliferation, and cell cycle arrest [10]. However, massive production of ROS during environmental stresses can damage cellular proteins and nucleic acids, as well as peroxidize lipids, which together can ultimately lead to cell death [11]. Previous research on the relationship between oxidative stress and aging indicated that oxidative stress is the origin of cellular senescence [12]. For example, the oxidative stressor hydrogen peroxide (H_2_O_2_) can induce an oxidative environment that rapidly leads to premature senescence [13].

The vascular endothelium comprises a thin layer of endothelium that lines the inner surface of blood vessels. Endothelial cells produce the vasorelaxant nitric oxide (NO) generated by NO synthase in endothelial cells (eNOS) that plays an important role in vasodilation. Aging of vascular endothelium in the elderly induces multifactorial dysfunctions in addition to reduced vasodilation [14] during the development of atherosclerosis, which is more pronounced with age [15, 16]. Thus, aging and endothelial cell dysfunction are closely related and insights into the mechanisms that are responsible for this dysfunction are important for delaying aging and enhancing the overall health of the elderly population.

Human umbilical vein endothelial cells (HUVECs) originate from the endothelium of human umbilical cord veins. HUVECs are used as a classic model system to study many aspects of endothelial function and disease, such as oxidative stress- and inflammation-related pathways in endothelia under normal and pathological conditions [17].

Coenzyme Q10 (CoQ_10_) is a ubiquitous lipid-soluble molecule found in many eukaryotic cells [18] that is essential for mitochondrial oxidative phosphorylation and electron transport chain activity [19]. With increasing age, CoQ10 concentrations in organisms decrease gradually and this decrease can be accompanied by the onset of physical dysfunction and the emergence of disease [20]. The critical role of CoQ_10_ in mitochondrial function [21] and its status as a lipid-soluble antioxidant have led to its use in therapeutic applications and clinical trials for CVD treatments [22]. Most CoQ_10_ in circulation and tissues exists in its reduced form (CoQ_10_H_2_), which acts as an antioxidant through its oxidation to an oxidized form (oxCoQ_10_) [23]. In addition, recent studies showed that the nonmitochondrial CoQ_10_-forming enzyme in Golgi membranes has specific cardiovascular protective functions that modulate eNOS activity through redox reactions involving CoQ_10_ [24]. NO synthetized by eNOS is known to be an essential factor for cardiovascular function in vertebrates [25] and prevents the progression of age-related dysfunction in endothelial cells [26]. Our previous studies showed that CoQ_10_H_2_ increases levels of cyclic adenosine monophosphate (cAMP) in tissues and cells and also enhances mitochondrial antioxidant function by activating SIRT1 and PGC-1*α* that in turn delays senescence and incidence of related diseases [27]. SIRT1 is recognized as an important deacetylase that is related to increasing eNOS-derived nitric oxide (NO) for prevention of endothelial senescence [28, 29]. In addition, we found that KKAy mice fed diets supplemented with CoQ_10_H_2_ reduced white adipose tissue and enhanced the function of brown adipose tissue by promoting the expression of SERCA2 and decreasing cytoplasmic Ca^2+^ levels in liver cells. These mice also had an enhanced fat metabolic rate through inhibition of the CaMKII-MEK1/2-ERK1/2 signaling pathway and increased cAMP levels [30]. In HUVECs, CoQ_10_H_2_ has been reported to have an anti-inflammatory function and to delay SASP acquisition in senescence status by attenuating miR-146a expression [31].

Here, our results showed that preincubation of HUVECs with CoQ_10_H_2_ prevented H_2_O_2_-induced premature senescence and ameliorated declines in physiological functions of endothelial cells. This action could be mediated by enhancing mitochondrial and endothelial function via the SIRT1-eNOS pathway and upregulating expression of antioxidant enzymes and decreasing intracellular ROS production.

## 2. Materials and Methods

### 2.1. Cell Culture

Human umbilical vein endothelial cells (HUVECs) were purchased from the Japanese Cancer Research Resources Bank (http://cellbank.nibiohn.go.jp/english/). The cells were grown in endothelial growth medium (EGM-2; Lonza Walkersville, MD, USA) at 37°C under a humidified atmosphere of 5% CO_2_, and the medium was changed every 2 days. HUVECs were passaged when they reached 80% confluence, and cells from passages 2–8 were used for all experiments. When HUVECs reached 90% confluence, the cells were divided into 4 experimental groups: (1) control group: untreated HUVECs; (2) CoQ_10_H_2_ group: cultured HUVECs incubated for 24 hours in medium with 10 *μ*M CoQ_10_H_2_ and then cultured for an additional 12 hours in medium lacking CoQ_10_H_2_; (3) H_2_O_2_ group: HUVECs cultured for 24 hours in medium without CoQ_10_H_2_ and cultured for another 12 hours in medium containing 100 *μ*M H_2_O_2_ and lacking CoQ_10_H_2_; and (4) CoQ_10_H_2_ + H_2_O_2_ group: HUVECs cultured for 24 hours in medium with 10 *μ*M CoQ_10_H_2_ and then cultured for 12 hours in medium with 100 *μ*M H_2_O_2_ and lacking CoQ_10_H_2_. We also incubated HUVECs in medium containing high glucose (30 mM; HG medium) to induce senescence. We pretreated HUVECs with 10 *μ*M CoQ_10_H_2_ and then cultured for an additional 72 hours in HG medium.

### 2.2. Real-Time RT-PCR

HUVECs were collected by scraping and the total RNA was extracted from cells using TRIzol Reagent (Invitrogen, CA) followed by treatment with DNA-Free (Applied Biosystems, CA) to remove contaminating DNA. Total RNA was subjected to reverse transcription using an Omniscript RT kit (Applied Biosystems, CA) with random primers (Applied Biosystems, CA). Quantitative real-time RT-PCR analysis was carried out using an ABI PRISM 7500 Sequence Detection System (Applied Biosystems, CA) with SYBR Green (Takara Bio, Tokyo, Japan). Primer sequences are listed in Table S1.

### 2.3. Senescence-Associated Galactosidase (SA-*β*-Gal) Staining

After 12 hours incubation in medium with or without H_2_O_2_, or 72 hours incubation in medium with or without HG, HUVECs were washed twice with PBS, fixed for 10 minutes with 10% formalin at room temperature, and washed twice in PBS. The cells were then incubated at 37°C for 12 hours with a staining solution (40 mM citric acid/sodium phosphate buffer pH 6.0, 0.5 mg/mL X-Gal, 5 mM potassium ferrocyanide, 5 mM potassium ferricyanide, 150 mM NaCl, and 2 mM MgCl_2_). SA-*β*-Gal-positive cells were observed by microscopy, and over 300 cells were counted in three independent fields to calculate the proportion of positive cells.

### 2.4. Analysis of Apoptosis

Apoptosis was assessed using an Annexin V-FITC Apoptosis Detection kit (Enzo Life Sciences, NW). HUVECs in each experimental group were washed with PBS after 12 hours incubation in medium with or without H_2_O_2_ and then collected by trypsinization. The collected cells were washed in PBS with gentle shaking and resuspended with 195 *μ*L of a specific binding buffer containing 5 *μ*L annexin V-FITC. After incubation for 10 minutes in the dark at room temperature, the cells were washed in PBS, resuspended in 190 *μ*L binding buffer, and then stained with 10 *μ*L propidium iodide (PI) (20 *μ*g/mL). Cellular fluorescence was analyzed with a FACSCanto II flow cytometer system (BD Biosciences, NJ), and the data were analyzed using BD FACSDiva™ software. HUVECs were classified as follows: normal healthy cells (Annexin V^−^/PI^−^), early apoptotic cells (Annexin V^+^/PI^−^), late apoptotic cells (Annexin V^+^/PI^+^), and necrotic cells (Annexin V^−^/PI^+^).

### 2.5. Total Reactive Oxygen Species (ROS) and Superoxide Production

Total ROS and superoxide production in HUVECs was determined using a total ROS/superoxide detection kit (Enzo Life Sciences, NW). HUVECs in each experimental group were washed with PBS after 12 hours incubation in medium with or without H_2_O_2_ and then stained according to the kit manufacturer's instructions. Then, cells were assessed by a FACSCalibur flow cytometer system (BD Biosciences, NJ) using the FL1 and FL2 channels to detect signals from ROS- and superoxide-sensitive reagents, respectively. We set the gate for 4 quadrants such that >99% of unstained untreated control HUVECs were grouped in the negative area. Over 89% of positive control cells (pyocyanin-treated) stained only with Oxidative Stress Detection Reagent (green) were gated into the ROS-positive area, and >89% of positive control cells (pyocyanin-treated) stained only with Superoxide Detection Reagent (orange) were gated into the superoxide-positive area. Each analysis was continued until 5000 cells were recorded. The obtained data were analyzed using BD CellQuest™ Pro software.

### 2.6. Measurement of Free Nitric Oxide (NO)

HUVECs were seeded directly into 12-well plates and after reaching 90% confluency, they were treated with CoQ_10_H_2_ and H_2_O_2_ as described above. NO production was measured by a ROS-ID NO Detection kit (Enzo Life Science, NW) on a LSM 5 EXCITER laser scanning microscope (Carl Zeiss Microscopy, Jena Deutschland) according to the manufacturer's instructions and analyzed with LSM Software ZEN 2009.

### 2.7. Analysis of Mitochondrial Membrane Potential

The probe 5,5′,6,6′-tetrachloro-1,1′,3,3′-tetraethylbenzimidazolylcarbocyanine iodide (JC-1) was used to measure mitochondrial membrane potential and depolarization in HUVECs. HUVECs were placed in 12-well plates, cultured to 90% confluence, and treated or not with CoQ_10_H_2_ and H_2_O_2_. Then the HUVECs were stained with a JC-1 Mitochondrial Membrane Potential Detection kit according to the manufacturer's instructions (Biotium Inc., CA). Green fluorescence (JC-1 as a monomer at low membrane potentials) and red fluorescence (JC-1 as “J-aggregates” at higher membrane potentials) were monitored with a LSM 5 laser scanning microscope (Carl Zeiss Microscopy, Jena Deutschland). Mitochondrial depolarization was indicated by a decrease in the red/green fluorescence intensity ratio.

### 2.8. Migration Assay

A scratch (wound healing) assay was performed to evaluate HUVEC migration activity. Treated HUVECs in 12-well plates were scratched with 200 *μ*L pipette tips. At 0 hour and 12 hours after scratching, images were taken under an inverted microscope to assess the ability of the cells to migrate into the wound area. The ratio of wound closure was calculated by analyzing images taken 0 and 12 hours after scratching with an image processing program (NIH ImageJ software, version 1.61) [32].

### 2.9. Cell Tube Formation Assay

Matrigel Matrix 356237 (Corning Inc., Life Sciences, MA) was coated on 15-well *μ*-angiogenesis slides at 10 *μ*L/well (ibidi GmbH, Planegg, Germany). The coated slides were incubated for 15 minutes at 37°C. Treated HUVECs (10,000 cells/well) were harvested and seeded into the Matrigel-containing wells and incubated for 6 hours at 37°C to allow tube formation. The wells were then imaged for capillary-like structures using an inverted microscope (Life Technologies). Quantification of the tubes was performed by taking 4 images of each chamber, which were then analyzed by ImageJ for in vitro angiogenesis [33].

### 2.10. Determination of Cellular Ca^2+^

Free cytosolic Ca^2+^ levels were determined with the fluorescent probe Fluo-3 (*Dōjindo* Laboratories), and the effect of CoQ_10_H_2_ on H_2_O_2_-induced changes in Ca^2+^ levels was monitored using real-time laser scanning confocal microscopy. Cells were cultivated in 35 mm plates and treated for 24 hours with vehicle (control) or 10 *μ*M CoQ_10_H_2_. The cells were then loaded for 30 minutes with 3 *μ*M Fluo-3, and medium was replaced with loading buffer to assess whether the CoQ_10_H_2_ effect was due to Ca^2+^ entry or release from intracellular stores. Cells were imaged with a LSM 7 laser scanning confocal microscope (Carl Zeiss Microscopy, Jena Deutschland), and the results were analyzed with LSM Software ZEN 2010. The baseline was established within the first minute of recording after which 100 *μ*L H_2_O_2_ was added to the plate. Epifluorescence images were then recorded. Results were analyzed with software accompanying the laser scanning confocal microscope. Results show the ratio of averaged profiles versus baseline for each treatment [34].

### 2.11. Analysis of the Concentration of CoQ_10_H_2_ and oxCoQ_10_

After incubation, cells were washed twice in PBS, pelleted, resuspended at 3 × 10^6^ in 700 *μ*L 2-propanol, frozen in acetone in dry ice, and stored on dry ice. For analysis, 150 *μ*L of 2-propanol containing an internal standard of ubiquinone-9 at 100 ng/mL was added to 200 *μ*L of the cell suspension, stirred with a vortex mixer for 30 seconds, and centrifuged for 5 minutes at 12,000 rpm. The supernatant was diluted 5-fold in 2-propanol : methanol (4 : 5, *v/v*), and 10 *μ*L of the diluted solution was injected into a LC/MS/MS system.

CoQ_10_H_2_ and oxCoQ_10_ levels in the cells were determined using a LC/MS/MS method described by Ruiz-Jimenez et al. [35] with a minor modification. Briefly, detection and quantification were performed using a QTRAP 5500 LC-MS/MS System (AB SCIEX, Framingham, MA, USA) equipped with a Turbo Ion Spray electrospray ionization (ESI) source and a Prominence UFLC system (Shimadzu, Kyoto, Japan). Chromatographic separation was performed on a YMC-UltraHT Pro C18 column, 50 mm × 2.0 mm I.D., 2.0 *μ*m particle size (YMC, Kyoto, Japan) maintained at 30°C. The mobile phase was methanol containing 5 mM ammonium formate : 2 -propanol : ultrapure water (50 : 47 : 3, *v/v/v*) pumped at a rate of 0.5 mL/minute. The run time was 5 minutes per injection. Calibration curves were derived from the peak area ratios (analyte/internal standard) using weighted linear least-squares regression of the peak area ratio versus the concentration of the standards. The limits of quantification of ubiquinol-10 and ubiquinone-10 were both 1.5 ng/10^6^ cells.

### 2.12. Statistical Analysis

All data are presented as means ± SD. Data were analyzed using one-way ANOVA followed by Tukey's test or Student's *t*-test using SPSS for Windows software (version 13.0; SPSS Inc., IL). *P* < 0.05 was considered to be statistically significant.

## 3. Results

### 3.1. Effect of CoQ_10_H_2_ on H_2_O_2_-Induced SA-*β*-Gal Activity and Senescence-Associated Gene Expression

HUVECs were incubated in medium containing different concentrations (0–30 *μ*M) of CoQ_10_H_2_ or oxCoQ_10_ for 24 hours, and the expression of *SIRT1* mRNA [27] was assessed by RT-PCR (Figure S1A–D). *SIRT1* mRNA levels were the highest with 10 *μ*M CoQ_10_H_2_. Next, HUVECs were incubated in medium containing 10 *μ*M CoQ_10_H_2_ or oxCoQ_10_ for different time periods (0–48 h). Under these conditions, expression levels of *SIRT1* mRNA were the highest after 24 hours incubation. Meanwhile, mRNA expression levels of *eNOS* and plasminogen activator inhibitor-1 (*PAI-1*) in HUVECs first treated with different concentrations of H_2_O_2_ (0–100 *μ*M) for 1 hour decreased and increased, respectively, in a dose-dependent manner (Figure S1E). Cell viability markedly decreased during incubation in medium containing 100 *μ*M H_2_O_2_ (0–48 hours), but preincubation with 10 *μ*M CoQ_10_H_2_ for 24 hours significantly promoted cell viability at 12 hours (Figure S1F). We used a 24-hour preincubation with 10 *μ*M CoQ_10_H_2_ followed by a 12-hour incubation with 100 *μ*M H_2_O_2_ for subsequent experiments. Notably, 10 *μ*M is almost the same as the plasma concentration in individuals who take oral CoQ10 supplements [36]. We observed that H_2_O_2_ treatment of HUVECs strongly decreased cell proliferation as evidenced by a reduction in the number of H_2_O_2_-treated cells relative to control group cells per field of vision. This effect could be prevented by preincubation with CoQ_10_H_2_ (Figure S2).

**Figure 1:**
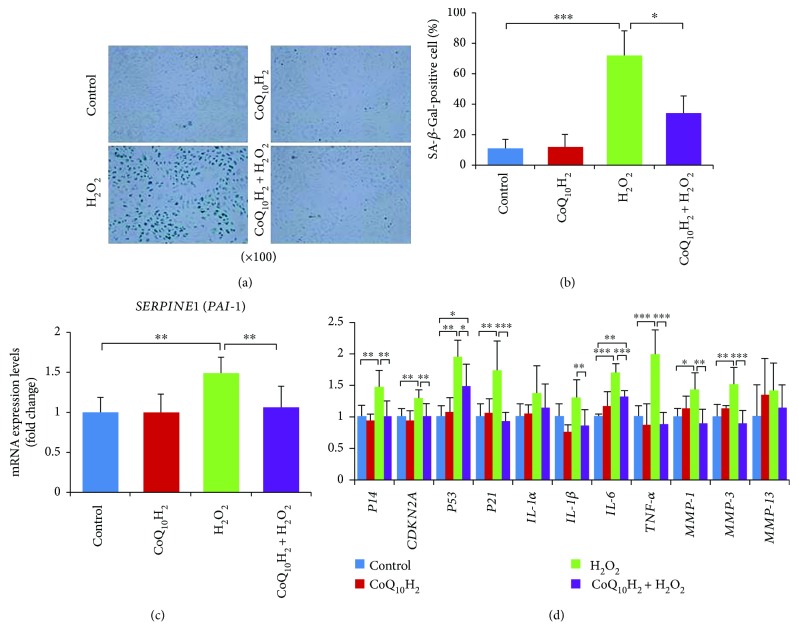
Preincubation with CoQ_10_H_2_ prevented H_2_O_2_-induced senescence of HUVECs. (a) Representative images of SA-*β*-Gal-stained cells from each group (control, CoQ_10_H_2_, H_2_O_2_, and CoQ_10_H_2_ + H_2_O_2_). (b) Percentage of SA-*β*-Gal-positive cells (*n* = 4). (c) Expression levels of PAI-1 mRNA (*n* = 10). (d) Expression of genes involved in the senescence-associated secretory phenotype. Histograms show fold change in mRNA level relative to control cells (*n* = 6–9). ^∗^ *P* < 0.05, ^∗∗^ *P* < 0.01, and ^∗∗∗^ *P* < 0.001; one-way ANOVA followed by Tukey's test.

The SA-*β*-Gal activity and *PAI-1* mRNA expression rate were detected to examine the senescent phenotype of HUVECs (Figures 1(a) to 1(c)). After treatment with H_2_O_2_ for 12 hours, about 72% of the cells were SA-*β*-Gal-stain positive in the H_2_O_2_ group but only 33% of the cells were positive in the CoQ_10_H_2_ + H_2_O_2_ group (Figures 1(a) and 1(b)). Increases in the mRNA expression levels of *PAI-1* in the H_2_O_2_ group were prevented in the CoQ_10_H_2_ + H_2_O_2_ group (Figure 1(c)). RT-PCR analysis of SASP-related gene expression (*P14*, *p16^INK4a^/CDKN2A*, *P53*, *P21*, *IL-1α*, *IL-1β*, *IL-6*, *TNF-α*, *MMP-1*, *MMP-3*, and *MMP-13*) showed that most genes, including *p14*, *p16 (CDKN2A)*, *P53*, *P21*, *IL-6*, *TNF-α*, *MMP-1*, and *MMP-3*, had increased expression after treatment with 100 *μ*M H_2_O_2_, but these adverse effects of H_2_O_2_ could be rescued upon preincubation with CoQ_10_H_2_ (Figure 1(d)).

### 3.2. Effect of CoQ_10_H_2_ on HG-Induced SA-*β*-Gal Activity and Senescence-Associated Gene Expression

**Figure 2:**
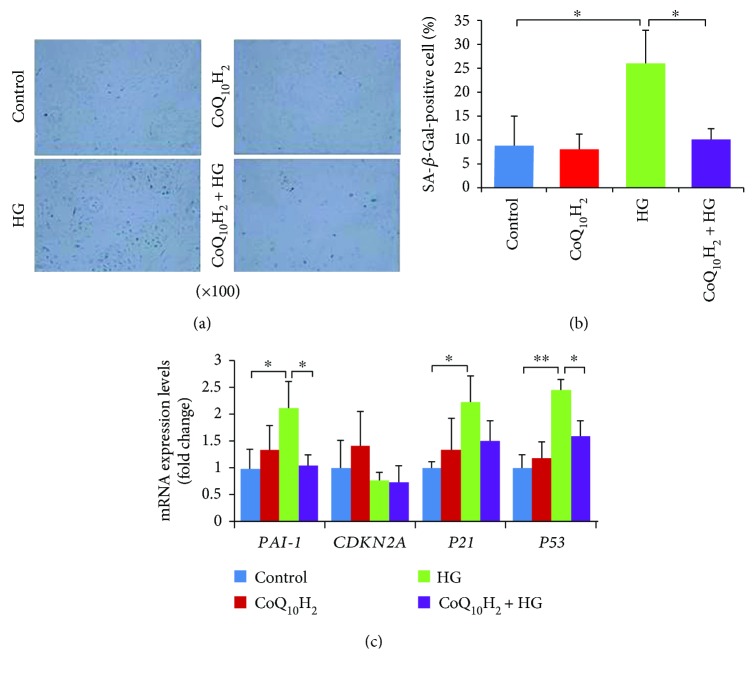
Preincubation with CoQ_10_H_2_ prevented HG-induced senescence of HUVECs. (a) Representative images of SA-*β*-Gal-stained cells from each group (control, CoQ_10_H_2_, HG, and CoQ_10_H_2_ + HG). (b) Percentage of SA-*β*-Gal-positive cells (*n* = 3). (c) Expression levels of senescence-associated mRNA and histograms show fold change in mRNA level relative to control cells (*n* = 3). ^∗^ *P* < 0.05, ^∗∗^ *P* < 0.01; one-way ANOVA followed by Tukey's test.

SA-*β*-Gal activity was detected to examine the HG-induced senescent phenotype of HUVECs [37, 38] (Figures 2(a) and 2(b)). After treatment with HG for 72 hours, ~26% of the cells were SA-*β*-Gal-stain positive in the HG group but only 10% of the cells were positive in the CoQ_10_H_2_ + HG group. Then, senescence-associated gene expression was detected and the results showed that several genes, including *PAI-1*and *P53*, had increased expression after treatment with 30 mM glucose (HG), but the adverse effects of HG could be rescued by preincubation with CoQ_10_H_2_ (Figure 2(c)).

### 3.3. CoQ_10_H_2_ Prevented H_2_O_2_-Induced Apoptosis and Necrosis

**Figure 3:**
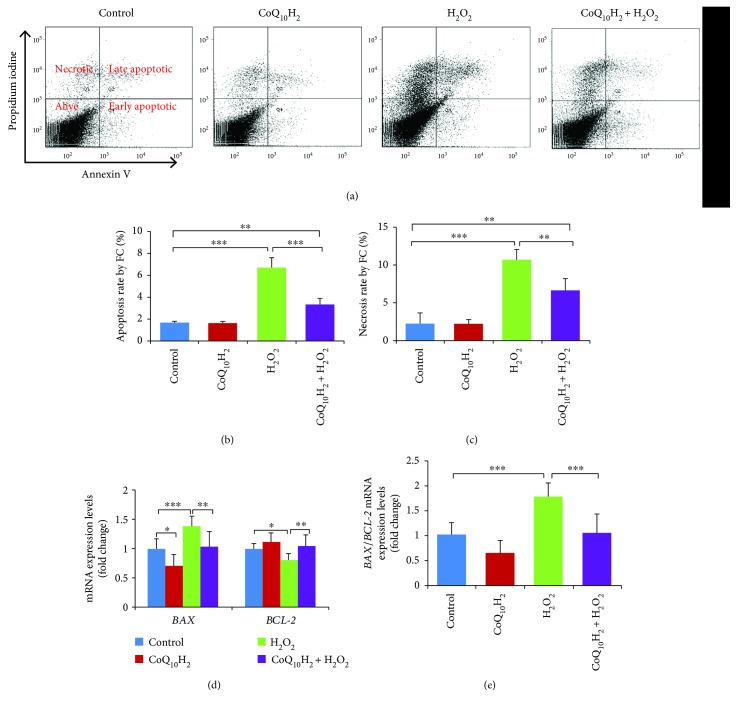
Preincubation with CoQ_10_H_2_ protects HUVECs from H_2_O_2_-induced apoptosis and necrosis. (a) Apoptotic cells were stained with annexin V-FITC and PI and evaluated by flow cytometry. Representative graphs of flow cytometric outputs for each group are shown. (b and c) Bar diagram representing apoptotic and necrotic cell populations (*n* = 6). (d) Analysis of *BAX* and *BCL-2* gene expression. Histograms show fold change in mRNA level relative to control cells (*n* = 9). (e) Histograms show *BAX*/*BCL-2* ratio (*n* = 9). ^∗^ *P* < 0.05, ^∗∗^ *P* < 0.01, and ^∗∗∗^ *P* < 0.001; one-way ANOVA followed by Tukey's test.

H_2_O_2_ was previously reported to promote endothelial tissue injury by inducing cell apoptosis and necrosis [39]. Here, we explored the effect of H_2_O_2_ on apoptosis and necrosis in HUVECs. Incubation of HUVECs with H_2_O_2_ for 12 hours increased the percentage of apoptosis-positive cells from 1.65% to 6.73%, indicating a modest but significant increase in apoptosis. Meanwhile, the percentage of apoptotic HUVECs preincubated with CoQ_10_H_2_ followed by incubation with H_2_O_2_ was below that for control cells (Figures 3(a) and 3(b)). Treatment with H_2_O_2_ increased the necrosis rate of HUVECs to 9%, but preincubation with CoQ_10_H_2_ could in part rescue H_2_O_2_-induced cell necrosis (Figure 3(c)). Relative to control cells, levels of mRNA for proapoptotic *BAX* were increased in the H_2_O_2_ group and decreased in the CoQ_10_H_2_ + H_2_O_2_ group. mRNA expression of antiapoptotic *BCL-2* decreased in the H_2_O_2_ group, whereas the levels in the CoQ_10_H_2_ + H_2_O_2_ group were similar to control cells and cells incubated with CoQ_10_H_2_ alone (Figure 3(d)). The *BAX/BCL-2* ratio dramatically increased in the H_2_O_2_ group but not in the CoQ_10_H_2_ + H_2_O_2_ group (Figure 3(e)). Preincubation with CoQ_10_H_2_ also inhibited an increase in levels of free cytosolic Ca^2+^ induced by H_2_O_2_ treatment of HUVECs (Figure S3).

### 3.4. CoQ_10_H_2_ Inhibited H_2_O_2_-Induced ROS Production

**Figure 4:**
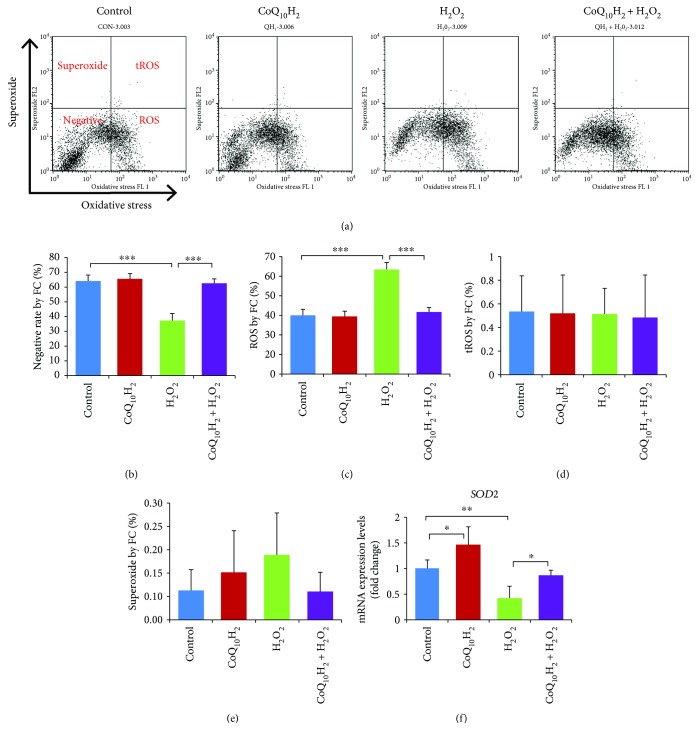
Preincubation with CoQ_10_H_2_ decreased H_2_O_2_-induced ROS production in HUVECS. (a) Cells were stained with two color oxidative stress detection reagents for determining ROS production and superoxide levels. Representative pictures of flow cytometry output show the cell population in the four fractions separated by ROS and superoxide (*n* = 6). (b, c, d, and e) Histograms show percentage of cells in negative fractionation, ROS-positive fraction, double-positive fraction, and superoxide-positive fraction. (f) Real-time RT-PCR analysis of *SOD2* mRNA expression in cells. Histograms show fold change in mRNA level relative to control cells (*n* = 6). ^∗^ *P* < 0.05, ^∗∗^ *P* < 0.01, and ^∗∗∗^ *P* < 0.001; one-way ANOVA followed by Tukey's test.

Next, we used flow cytometry to examine the effect of CoQ_10_H_2_ and H_2_O_2_ on intracellular ROS production in HUVECs (Figure 4(a)). Treatment with H_2_O_2_ decreased the percentage of ROS/superoxide-negative cells compared with the control group from 64.44% to 37.48%, whereas 24 hours preincubation with CoQ_10_H_2_ prevented this decrease such that the values were similar to that of control cells. Treatment with H_2_O_2_ induced the production of ROS, which was prevented by preincubation with CoQ_10_H_2_. However, the percentage of superoxide-positive cells and total ROS (tROS) was not affected by H_2_O_2_ treatment (Figures 4(b) and 4(e)), although preincubation with CoQ_10_H_2_ did increase mRNA expression of the ROS scavenger enzyme *SOD2* compared with the control group (Figure 4(f)). Meanwhile, treatment with H_2_O_2_ resulted in significant downregulation of *SOD2* mRNA expression, which was significantly relieved by preincubation with CoQ_10_H_2_.

### 3.5. CoQ_10_H_2_ Prevented a Decrease in NO Production Induced by H_2_O_2_

**Figure 5:**
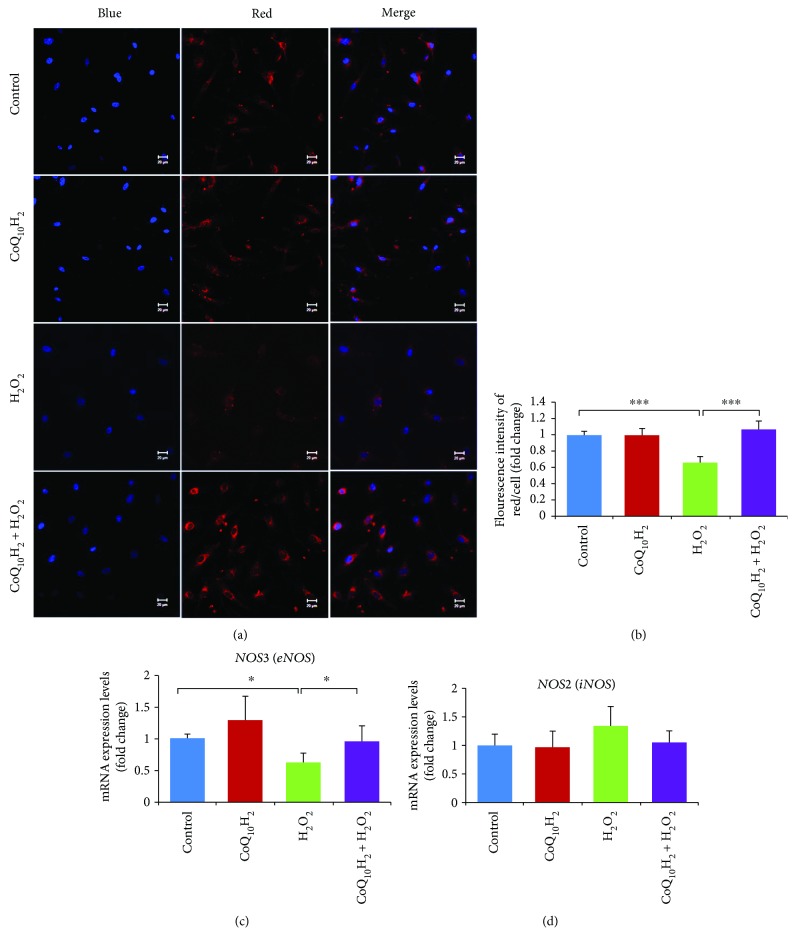
Preincubation with CoQ_10_H_2_ prevents H_2_O_2_-induced suppression of NO production in HUVECs. (a) Representative images of staining with DAPI (blue) and a NO-specific fluorometric probe (red) acquired using a laser scanning microscope. The two images in each row were captured within the same field and then merged. (b) The intracellular NO level was calculated from the fluorescence intensity in each cell. Histograms show fold change in NO levels relative to control cells (*n* = 6). (c and d) Analysis of *eNOS* and *iNOS* gene expression. Histograms show fold change in mRNA level relative to control cells (*n* = 10). ^∗^ *P* < 0.05 and ^∗∗∗^ *P* < 0.001; one-way ANOVA followed by Tukey's test.

NO production is an important feature of endothelial cell function. Although laser scanning confocal microscopy showed weaker (~44%) NO-specific red fluorescent signals in the cytosol of H_2_O_2_-treated HUVECs compared with the control group, preincubation with CoQ_10_H_2_ increased red fluorescent signals to similar levels as that for the control group (Figures 5(a) and 5(b)). H_2_O_2_ treatment also reduced levels of *eNOS* mRNA compared with the control group, whereas preincubation with CoQ_10_H_2_ resulted in a significant upregulation of *eNOS* mRNA compared with the H_2_O_2_-treated group (Figure 5(c)), but treatment with either CoQ_10_H_2_ or H_2_O_2_ did not affect the level of *iNOS* mRNA compared to the control cells or cells treated with CoQ_10_H_2_ alone (Figure 5(d)).

### 3.6. Effect of CoQ_10_H_2_ on H_2_O_2_-Dependent Reductions in Mitochondrial Membrane Potential

**Figure 6:**
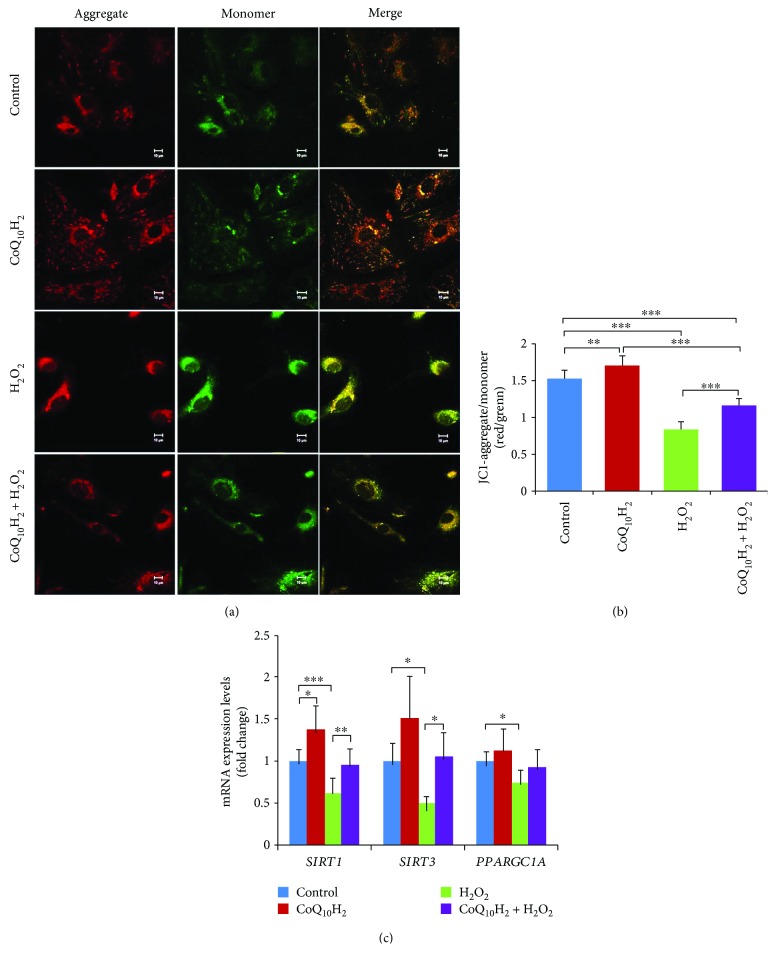
Preincubation with CoQ_10_H_2_ prevents H_2_O_2_-induced deterioration of mitochondrial membrane potential in HUVECs. (a) Representative laser scanning microscopy images of aggregated (red) and monomeric (green) JC-1. The two images in each row were captured within the same field and then merged. (b) Mitochondrial depolarization was demonstrated by a change in JC-1 fluorescence from red to green (aggregate/monomer) (*n* = 6). (c) Real-time RT-PCR analysis of *SIRT1*, *SIRT3*, and *PGC-1α (PPARGC1A)* mRNA expression. Histograms show fold change in mRNA level relative to the control cells (*n* = 6–9). ^∗^ *P* < 0.05, ^∗∗^ *P* < 0.01, ^∗∗∗^ *P* < 0.001; one-way ANOVA followed by Tukey's test.

We next analyzed HUVECs in each group using the mitochondrial membrane potential-sensitive dye JC-1. In control cells and cells that were preincubated with CoQ_10_H_2_, JC-1 aggregates (red fluorescence) in the cytosol were dispersed, whereas in cells exposed to H_2_O_2_, green fluorescence, indicative of the monomeric form of JC-1, was more prominent (Figure 6(a)). Moreover, HUVECs pretreated with CoQ_10_H_2_ displayed a significant upregulation in the ratio of red/green fluorescence relative to the control group, whereas reductions in the ratio of red/green fluorescence seen for H_2_O_2_-treated cells indicated a deterioration in mitochondrial membrane polarization. Preincubation with CoQ_10_H_2_ reduced these H_2_O_2_-dependent decreases in mitochondrial membrane potential (Figure 6(b)). To test whether the CoQ_10_H_2_-dependent preservation of mitochondrial membrane potential was associated with upregulated expression of genes involved in mitochondrial function, we analyzed *SIRT1*, *SIRT3*, and *PGC-1α* (*PPARGC1A*) mRNA levels in each group. H_2_O_2_ treatment reduced *SIRT1*, *SIRT3*, and *PGC-1α* mRNA levels compared with the control group, whereas cells preincubated with CoQ_10_H_2_ prior to H_2_O_2_ treatment showed significant upregulation of *SIRT1* and *SIRT3* mRNA relative to cells treated with H_2_O_2_ alone (Figure 6(c)).

### 3.7. CoQ_10_H_2_ Restored Migration Activity and Tube Formation Inhibited by H_2_O_2_ or HG Treatment

**Figure 7:**
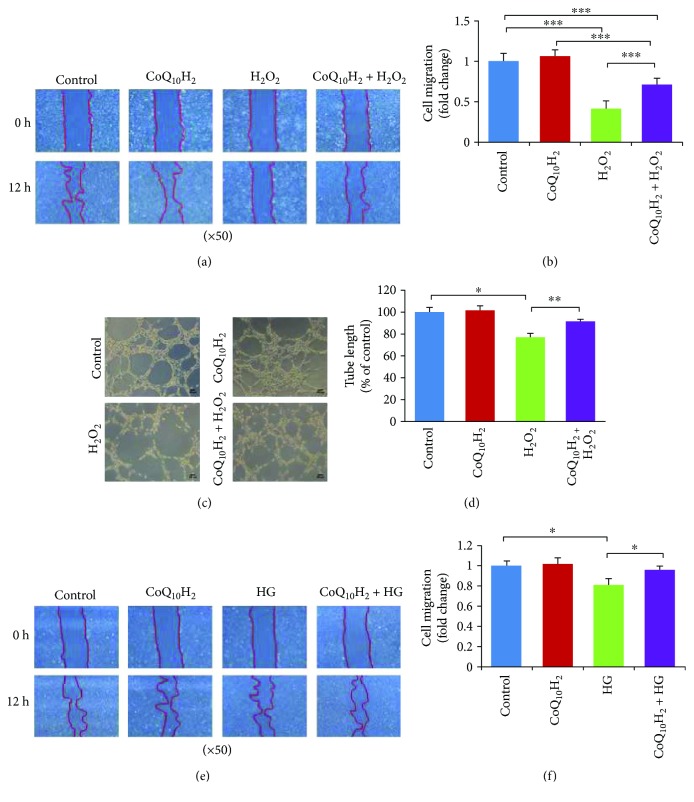
Preincubation with CoQ_10_H_2_ prevented H_2_O_2_ or HG-induced reduction in migration and tube formation by HUVECs. (a) Representative images of cell migration analysis evaluated using a wound-healing assay conducted over 12 hours in H_2_O_2_-induced reduction of migration. (b) Histograms show fold change in migration activity relative to control cells (*n* = 9). (c) Representative images from a H_2_O_2_-induced tube formation assay after a 6-hour incubation. (d) Histograms show fold change in total cell tube length relative to control cells (*n* = 6). (e) Representative images of cell migration over 12 hours evaluated using a wound-healing assay in HG-induced reduction of migration. (f) Histogram shows fold change in migration activity relative to control cells (*n* = 3). ^∗^ *P* < 0.05, ^∗∗^ *P* < 0.01, and ^∗∗∗^ *P* < 0.001; mean ± SD, one-way ANOVA followed by Tukey's test.

Cell migration and tube formation are involved in angiogenesis, so we next assessed the effect of CoQ_10_H_2_ treatment on impairments of these functions in HUVECs treated with 100 *μ*M H_2_O_2_. H_2_O_2_ treatment of HUVECs strongly decreased the migration activity of these cells as evaluated by a standard cell migration assay (Figures 7(a) and 7(b)). Similarly, treatment with H_2_O_2_ resulted in a 30% reduction in capillary-like tube formation by HUVECs; both of these effects could be prevented by preincubation with CoQ_10_H_2_ (Figures 7(c) and 7(d)). We also observed a similar effect of CoQ_10_H_2_ treatment in HUVECs treated with 60 *μ*M H_2_O_2_ (Figure S4).

We next assessed the effect of CoQ_10_H_2_ treatment on HG-induced impairment of cell migration function in HUVECs. HG treatment of HUVECs evidently decreased the migration activity of these cells as evaluated by a standard cell migration assay, and this reduction could be rescued by preincubation with CoQ_10_H_2_ (Figures 7(e) and 7(f)).

There was no protective effect of incubating cells with 10 *μ*M CoQ_10_H_2_ for 24 hours after treatment with 100 *μ*M H_2_O_2_ for 12 hours (Figures S5A and B). However, when the incubation time for 100 *μ*M H_2_O_2_ was shortened to 3 hours, postincubation with CoQ_10_H_2_ did promote a significant protective effect (Figures S5C and D).

### 3.8. CoQ_10_H_2_ and oxCoQ_10_ Concentrations in CoQ_10_H_2_- or H_2_O_2_-Treated HUVECs

**Figure 8:**
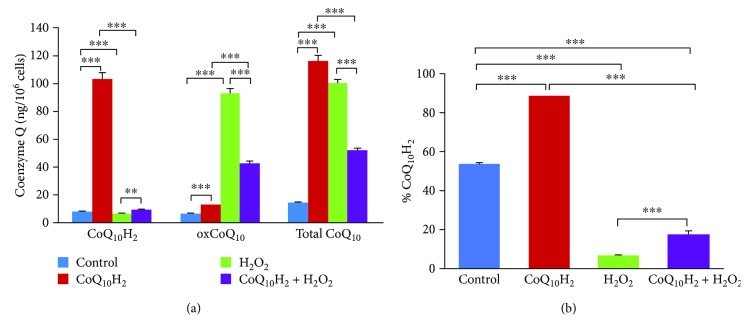
Intracellular CoQ_10_H_2_ and oxCoQ_10_ levels in HUVECs changed dramatically after treatment with H_2_O_2_ and CoQ_10_H_2_. (a) The amount of reduced (CoQ_10_H_2_) and oxidized (oxCoQ_10_) forms of coenzyme Q10 in whole cell extracts was determined. (b) The percentage of CoQ_10_H_2_ in total coenzyme Q10 (Total CoQ_10_) was calculated (*n* = 4). ^∗∗^ *P* < 0.01, ^∗∗∗^ *P* < 0.001; one-way ANOVA followed by Tukey's test.

Incubation of HUVECs in medium containing 10 *μ*M CoQ_10_H_2_ led to small and dramatic increases in the intracellular levels of CoQ_10_H_2_ and oxCoQ_10_, respectively (Figure 8(a)), and the percentage of CoQ_10_H_2_ relative to total CoQ_10_ was increased compared with the control group (Figure 8(b)). Interestingly, the concentration of oxCoQ_10_ was markedly increased in H_2_O_2_-treated cells compared with the control group. However, CoQ_10_H_2_ preincubation prevented the increase in oxCoQ_10_ and increased the ratio of CoQ_10_H_2_ to oxCoQ_10_ (CoQ_10_H_2_/oxCoQ_10_) in the CoQ_10_H_2_ + H_2_O_2_-treated group.

We also analyzed H_2_O_2_-induced changes in CoQ_10_H_2_ and oxCoQ_10_ concentrations. HUVECs were treated with 100 *μ*M H_2_O_2_ for various times (0–24 hours), and cellular CoQ_10_H_2_ and oxCoQ_10_ levels were determined for each time point. The oxCoQ_10_ levels in H_2_O_2_-treated HUVECs increased in a time-dependent manner, while the percentage of CoQ_10_H_2_ in the total coenzyme Q10 (Total CoQ_10_) decreased (Figures S6A and B). The mRNA expressions of the CoQ_10_ biosynthesis genes *PDSS2* and *COQ_2_* were both markedly increased in H_2_O_2_-treated HUVECs after 12 hours compared with the 0-hour group (Figure S6C). The cell number and protein concentration of HUVECs per culture flask were decreased for the 12-hour group relative to the 0-hour group, but the changes were not significant (Figure S6D).

## 4. Discussion

The main findings of our study are that CoQ_10_H_2_ prevented functional damage of endothelial cells that accompany premature senescence caused by oxidative stress. Specifically, we examined the effect of preincubation with CoQ_10_H_2_ prior to H_2_O_2_ exposure on cell senescence, cell proliferation, apoptosis, and oxidative damage as well as how these compounds affected various endothelial cell functions, including eNOS production, mitochondrial activity and migration, and tube formation activities. In addition, we assessed the effect of preincubation with CoQ_10_H_2_ prior to HG exposure on cell senescence.

In addition to its bioenergetic role as a proton carrier in the mitochondrial respiratory chain, CoQ_10_H_2_ has a protective role by inhibiting lipid peroxidation in membranes and lipoproteins [19, 40]. Oxidative stress and a proinflammatory state are major characteristics of aging and many age-related disorders, including CVD [41]. During aging of humans and animals, levels of oxidative stress increase, whereas the capacity of antioxidant defense systems and levels of CoQ_10_ decline [20, 42]. CoQ_10_ supplementation has beneficial effects for both aging subjects and CVD patients by enhancing endothelial function [43]. At a cellular level, the antioxidant effect of CoQ_10_ on endothelial functions is thought to be mediated through protection against mitochondrial dysfunction [44], although the details of these mitochondria-related mechanisms are unclear, and mitochondria may not be the only cellular site that is affected by CoQ10. Indeed, nonmitochondrial effects of CoQ_10_ in endothelial cells include regulating NO production and signaling in the Golgi compartment mediated through altered eNOS activity and membrane redox status [24]. eNOS is known to be a critical regulator of cardiovascular functions through its generation of NO, which can slow the progression of endothelial cell senescence and dysfunction [26, 45]. During aging, NO production by human endothelial cells does decrease [46]. Meanwhile, mRNA expression levels of *PAI-1*, which is associated with atherosclerosis, and SA-*β*-Gal activity, a marker of senescence, were increased in senescent endothelial cells and aortas from aged mice, respectively [47]. H_2_O_2_ can induce premature senescence and apoptosis in endothelial cells [48, 49], and H_2_O_2_ acts as an oxidative stressor to modulate levels of endogenous oxidants and ROS products [50, 51].

Our previous studies revealed that dietary supplementation with the reduced form of CoQ_10_H_2_ could effectively improve mitochondrial functions, inhibit oxidative stress in the liver, and decelerate senescence in senescence-accelerated mice (SAMP1 strain) [27]. Subsequently, we also found that dietary supplementation with CoQ_10_H_2_ of KKAy mice reduced the amount of white adipose tissue and enhanced the function of brown adipose tissue by promoting lipid metabolism [30]. In these studies, we showed that CoQ_10_H_2_ decreases cytoplasmic Ca^2+^ in liver cells by enhancing activity of Ca^2+^ pumps in the endoplasmic reticulum, which in turn led to increased cAMP levels and enhanced activity of mitochondrial antioxidant defense systems via upregulated gene expression of *AMPK*, *SIRT1*, and *PGC1-α* signaling proteins [30]. Here, we determined the optimal concentration and times for preincubation with CoQ_10_H_2_ and incubation with H_2_O_2_ in HUVECs using *SIRT1* expression and cell viability, respectively, as indices.

The SASP includes expression of proinflammatory cytokines, tumor necrosis factors, and matrix metalloproteinases in body tissues and is a hallmark of biological and premature aging [52]. Cellular senescence progression is thought to be regulated by the tumor suppressor pathways p14ARF/p53/p21 and p16INK4a/retinoblastoma (Rb) [53, 54]. Treatment with H_2_O_2_ negatively impacts cellular functions as well induces senescence and apoptosis in various cells, including endothelial cells [48, 55]. CoQ10 has been reported to decrease expression of proinflammatory cytokines in replicative senescent HUVECs and p53, p21, and p16^INK4a^ expression in induced premature mesenchymal stem cells [31, 56]. Our results showed that preincubation of HUVECs with CoQ_10_H_2_ protected against senescence, apoptosis, and necrosis induced by H_2_O_2_ and also promoted rescue from ROS overproduction, mitochondria hypofunction, and diminished NO production. Furthermore, CoQ_10_H_2_-mediated inhibition of increased intracellular Ca^2+^ levels after H_2_O_2_ treatment (Figure S3) is consistent with our previous results obtained with hepatocarcinoma HepG2 cells [30].

Using the fluorescent dye JC-1 [57] to analyze functional damage of mitochondria by assessing changes in mitochondrial membrane potential (MMP), we found that pretreatment with CoQ_10_H_2_ could mitigate H_2_O_2_-induced reductions in MMP, which must be preserved to avoid apoptosis [58]. *SIRT1* is proposed to be a critical gene in aging and age-related diseases [59] that affects mitochondrial biogenesis through regulation of *PGC-1α* expression [60], whereas mitochondrial *SIRT3* activates mitochondrial antioxidant defense systems in the presence of caloric restriction (CR) [61]. Consistent with these earlier findings, here, we showed that CoQ_10_H_2_ enhanced activity of the mitochondrial antioxidative system in HUVECs.

Angiogenesis is the physiological process wherein new capillaries grow from existing vessels. Endothelial cell functions such as migration and tube formation play important roles in the formation of these vessel sprouts [62, 63]. Our results suggested that CoQ_10_H_2_ can enhance activity of microvascular endothelial cells to promote wound repair while also impairing formation of tube-like structure induced by oxidative stress.

CoQ_10_ is endogenously produced in all cells, and its reduced form (CoQ_10_H_2_) has important antioxidative effects [19, 40]. In this study, we detected dramatic increases in cellular CoQ_10_H_2_ concentrations following supplementation with CoQ_10_H_2_ in media for HUVEC culture, which is similar to previous findings [64]. Exposure of HUVECs to H_2_O_2_ resulted in a rapid increase in cellular oxCoQ_10_ (Figure 8, Figure S6A), and this effect was prevented in part by pretreatment with CoQ_10_H_2_ (Figure 8). We analyzed the mRNA expression of *PDSS2* and *COQ2* and the important enzymes for CoQ_10_ biosynthesis and found that the expression levels for both increased significantly (Figure S6C). Additional studies will be needed to fully characterize the effect of H_2_O_2_ on CoQ_10_ biosynthesis, which is known to involve at least 13 genes (e.g., *PDSS2*, *COQ1*, *COQ2*, and *COQ3*) [65]. Increased amounts of total CoQ_10_ induced by H_2_O_2_ treatment could indicate that oxidative damage promotes increased biosynthesis of oxCoQ_10_ and that CoQ_10_H_2_ supplementation curbs the effect of H_2_O_2_. The limited cellular incorporation of CoQ_10_ in the surviving cell population in the presence of oxidative stress induced by H_2_O_2_ treatment suggests that surviving cells may invoke an adaptive response mediated through upregulation of CoQ_10_ synthesis. However, increased amounts of oxCoQ_10_ might not translate to enhanced viability and functionality in light of the very high percentage of oxCoQ_10_ that could disrupt the optimal reduced state of CoQ_10_ in H_2_O_2_-exposed cells. Taken together, our results indicate that CoQ_10_H_2_ plays a critical role in cellular processes and CoQ_10_ synthesis, but further studies are needed to elucidate the mechanism and biological significance of the observed CoQ_10_ upregulation.

Finally, we selected the concentration of H_2_O_2_ (100 *μ*M) based on previous reports [48, 49]. We showed that 100 *μ*M H_2_O_2_ affected eNOS and PAI-1 mRNA expression in HUVECs and reduced cell viability to 20% (Figures S1E and F). To determine cell viability, we used the MTT method, which can provide information not only for cytotoxicity and cell proliferation but also for mitochondrial activation [66–68]. Thus, it is clear that the mechanism by which CoQ_10_H_2_ preincubation protects against the negative effects of H_2_O_2_ treatment is complex, and for a selected population of HUVECs, cellular senescence and mitochondrial function may also be involved. Additional investigations will be needed to characterize the detailed effects of CoQ_10_H_2_ on dysfunction induced by oxidative stress in endothelial cells.

In conclusion, H_2_O_2_ can induce HUVEC senescence through oxidative stress and increased ROS production. CoQ_10_H_2_ can markedly increase resistance to oxidative damage by enhancing mitochondrial function as well as prevent senescence and diminished function of endothelial cells treated with H_2_O_2_. Our results would facilitate another perspective for the investigation of the potential of CoQ_10_H_2_ to protect against age-associated exacerbation of CVD. *In vivo* studies will be needed to study how CoQ_10_H_2_ affects the development of endothelial dysfunction and the mechanisms by which CoQ_10_H_2_ regulates endothelial cell aging in both older individuals and CVD patients.

## Supplementary Materials

Supplementary Materials Table S1: primer sequences for real-time RT-PCR. Figure S1: H_2_O_2_, CoQ_10_H_2_, and oxCoQ_10_ affect HUVEC-related gene expression and CoQ_10_H_2_ prevents H_2_O_2_-mediated decreases in cell viability. Figure S2: preincubation with CoQ_10_H_2_ prevented H_2_O_2_-induced reduction of cell proliferation in HUVECs. Figure S3: CoQ_10_H_2_ blocks H_2_O_2_-induced increases in free cytosolic Ca^2+^ levels in endothelial cells. Figure S4: CoQ_10_H_2_ treatment prevented H_2_O_2_-induced reduction in migration and tube formation. Figure S5: CoQ_10_H_2_ affects H_2_O_2_-dependent reduction in endothelial cell migration. Figure S6: CoQ_10_H_2_ and oxCoQ_10_ levels in HUVECs increased after H_2_O_2_ treatment.
