# Supplementary material for: Coenzyme Q10 Prevents Senescence and Dysfunction Caused by Oxidative Stress in Vascular Endothelial Cells
Source: Oxid Med Cell Longev. 2018 Jul 8;2018:3181759. doi: 10.1155/2018/3181759 (PMC6079399; doi:10.1155/2018/3181759)
Supplement: Supplementary Materials — Table S1: primer sequences for real-time RT-PCR. Figure S1: H2O2, CoQ10H2, and oxCoQ10 affect HUVEC-related gene expression and CoQ10H2 prevents H2O2-mediated decreases in cell viability. Figure S2: preincubation with CoQ10H2 prevented H2O2-induced reduction of cell proliferation in HUVECs. Figure S3: CoQ10H2 blocks H2O2-induced increases in free cytosolic Ca2+ levels in endothelial cells. Figure S4: CoQ10H2 treatment prevented H2O2-induced reduction in migration and tube formation. Figure S5: CoQ10H2 affects H2O2-dependent reduction in endothelial cell migration. Figure S6: CoQ10H2 and oxCoQ10 levels in HUVECs increased after H2O2 treatment. [file 3181759.f1.pdf]

Coenzyme Q10 prevents senescence and dysfunction caused by oxidative stress in vascular endothelial cells.

Manuscript ID: 3181759.v2

Supplemental Information

Expanded Materials and Methods

Effects of H_2O_2, $CoQ_{10}H_2$ and $oxCoQ_{10}$ treatments on HUVECs

HUVECs were incubated for 24 hours in medium containing different concentrations of $CoQ_{10}H_2$ or $oxCoQ_{10}$ (0 - 30 μM) or with 10 μM $CoQ_{10}H_2$ or $oxCoQ_{10}$ for different time periods (0 - 48 hours). HUVECs were also treated with different concentrations of H_2O_2 (0 - 100 μM) for 1 hour. Total RNA was then extracted from the cells and subjected to reverse transcription with random primers. RT-PCR analysis was then performed.

Cell viability analysis

Cell viability was assessed using the 3-(4,5-dimethylthiazol-2-yl)-2,5-diphenyltetrazolium bromide (MTT) assay. In brief, cells were seeded in 96-well flat-bottomed plates and incubated for 24 hours in medium with or without 10 μM $CoQ_{10}H_2$. The medium was then removed and the HUVECs were washed twice with phosphate-buffered saline (PBS) before H_2O_2 stimulation. At each time point (0 - 48 hours), the culture medium was replaced with fresh medium containing 0.5 mg/ml MTT, and cells were incubated in a CO_2 incubator at 37 °C for 4 hours. Absorbance at 570 nm was measured using a plate reader.

Cell proliferation analysis

In brief, treated HUVECs (1×10^5 cells/well) were harvested and seeded into 12-well plates and incubated for 48 hours at 37 ℃. To examine cell proliferation the wells

were then imaged with an inverted microscope (Life Technologies). Cell counts were made for 3 independent images from randomly selected wells.

Migration assay by pre-stimulated H_2O_2

The pre-stimulated by H_2O_2 experimental group: (1) control group: untreated HUVECs; (2) $CoQ_{10}H_2$ group: cultured HUVECs incubated with 10 μM $CoQ_{10}H_2$ for 24 hours; (3) H_2O_2 group: cultured HUVECs incubated with 100 μM H_2O_2 alone for 12 hours or 3 hours; (4) Treated group: cultured HUVECs pre-stimulated with 100 μM H_2O_2 for 12 hours or 3 hours and then incubated with 10 μM $CoQ_{10}H_2$ for 24 hours. To evaluate cell migration, scratch assays was performed wherein confluent, treated HUVECs in 12-well plates were scratched with 200 μl pipette tips. At 0 hour and 12 hours after scratching, images were taken using an inverted microscope to assess cell migration into the wound area. The ratio of wound closure was calculated by measuring each image with Image J.

Assay of migration and tube formation following H_2O_2 treatment

The experimental group: (1) control group: untreated HUVECs; (2) $CoQ_{10}H_2$ group: cultured HUVECs incubated for 24 hours in medium with 10 μM $CoQ_{10}H_2$ and then cultured for an additional 12 hours in medium lacking $CoQ_{10}H_2$; (3) H_2O_2 group: HUVECs cultured for 24 hours in medium without $CoQ_{10}H_2$ and cultured for another 12 hours in medium containing 60 μM H_2O_2 and lacking $CoQ_{10}H_2$; (4) $CoQ_{10}H_2$ + H_2O_2 group: HUVECs cultured for 24 hours in medium with 10 μM $CoQ_{10}H_2$ for 24 hours and then cultured for 12 hours in medium with 60 μM H_2O_2 and lacking $CoQ_{10}H_2$. The ratio of wound closure and quantification of tubes was calculated for each image with Image J.

Analysis of the H_2O_2-induced changes in $CoQ_{10}H_2$ and oxCoQ_{10} concentration and expression of genes involved in CoQ_{10} biosynthesis

In brief, cells were seeded in culture flasks and incubated until HUVECs reached

90% confluence. The medium was then removed and the HUVECs were washed twice with PBS and then treated with 100 μM H_2O_2. At each time point (0 - 24 hours), cellular $CoQ_{10}H_2$ and oxCoQ_{10} levels were determined using a LC/MS/MS method described by Ruiz-Jiménez et al. The mRNA expression of *PDSS2* and *COQ_2*, which are both involved in CoQ_{10} biosynthesis, in the 0 and 12 hours group was detected in HUVECs using real-time PCR.

Cell number and cell protein concentration assay

HUVECs were first trypsinized after treatment with 0 or 100 μM H_2O_2 for 12 hours. To assess cell numbers, cells resuspended in growth medium were diluted 1:1 with 0.4% trypan blue stain from Gibco™ (Invitrogen Corporation, USA). Stained and unstained cells were counted using a Luna™ Automated Cell Counter (Logos Biosystems, USA). To assess protein content, whole-cell extracts after were prepared from H_2O_2-treated HUVECs lysed at 4 ℃ in cell lysis buffer (Cell Signaling Technology, MA) supplemented with protease inhibitors (Sigma Aldrich, MO). Protein samples were centrifuged at 15,000 g for 10 minutes and the supernatants were collected for determination of protein concentrations using the BCA protein Assay Kit (Thermo Fisher Scientific, CO).

Supplemental Tables and Figure Legends

Table S1. Primer sequences for Real-time RT-PCR

Gene	Forward (5' - 3')	Reverse (5' - 3')
SIRT1	GCGGGAATCCAAAGGATAAT	GCACCTAGGACATCGAGGAA
SIRT3	GTCGGGCATCCCTGCCTCAAAGC	GGAACCCTGTCTGCCATCACGTCAG
SOD2	GCAGAAGCACAGCCTCCCCG	CCTTGGCCAACGCCTCCTGG
PGC-α	GTGAAGACCAGCCTCTTTGC	TCACGTCTCCATCTGTCAGC
PAI-1	AGCTCCTTGTACAGATGCCG	ACAACAGGAGGAGAAACCCA
eNOS	GAAACGGTCGCTTCGACGT	ATCCCACCCAGTCAATCCCT
iNOS	CCTGAGCTCTTCGAAATCCCA	CCCGAAACCACTCGTATTTGG
P16 ^{INK4a}	AGCCTTCGGCTGACTGGCTGG	CTGCCCATCATCATGACCTGGA
P21	ATGTCCGTCAGAACCCATG	CAGTGGTGTCTCGGTGAC
P53	CTCCTCTCCCCAGCCAAAGA	GGAACATCTCGAAGCGCTCA
P14^{arf}	GTGGCCCTCGTGCTGATG	AGCACCACCAGCGTGTCC
BCL2	CTTTGAGTTCGGTGGGGTCA	GGGCCGTACAGTTCCACAAA
BAX	CCGCCGTGGACACAGAC	CAGAAAACATGTCAGCTGCCA
IL-1α	GTCTCTGAATCAGAAATCCTTCTATC	CATGTCAAATTTCACTGCTTCATCC
IL-1β	GGATATGGAGCAACAAGTGG	ATGTACCAGTTGGGGAACTG
IL-6	GCCTTCGGTCCAGTTGCCTT	AGTGCCTCTTTGCTGCTTTCAC
TNF-α	CGGGACGTGGAGCTGGCCGAGGAG	CACCAGCTGGTTATCTCTCAGCTC
MMP-1	GGGGCTTTGATGTACCCTAGC	TGTCACACGCTTTTGGGGTTT
MMP-3	CGGTTCCGCCTGTCTCAAG	CGCCAAAAGTGCCTGTCTT
MMP-13	GGACAAGTAGTTCCAAAGGCTACAA	CTTTTGCCGGTGTAGGTGTAGATAG
PDSS2	GAATCAGGTAGTGTCAGAGG	GAGGCTATTCCAGCTGTCATG
COQ2	TATGATACTATTTATGCCCA	GCTCAGTGCCCCCAGCATTG
β-Actin	TGGCACCCAGCACAATGAA	CTAAGTCATAGTCCGCCTAGAAGCA

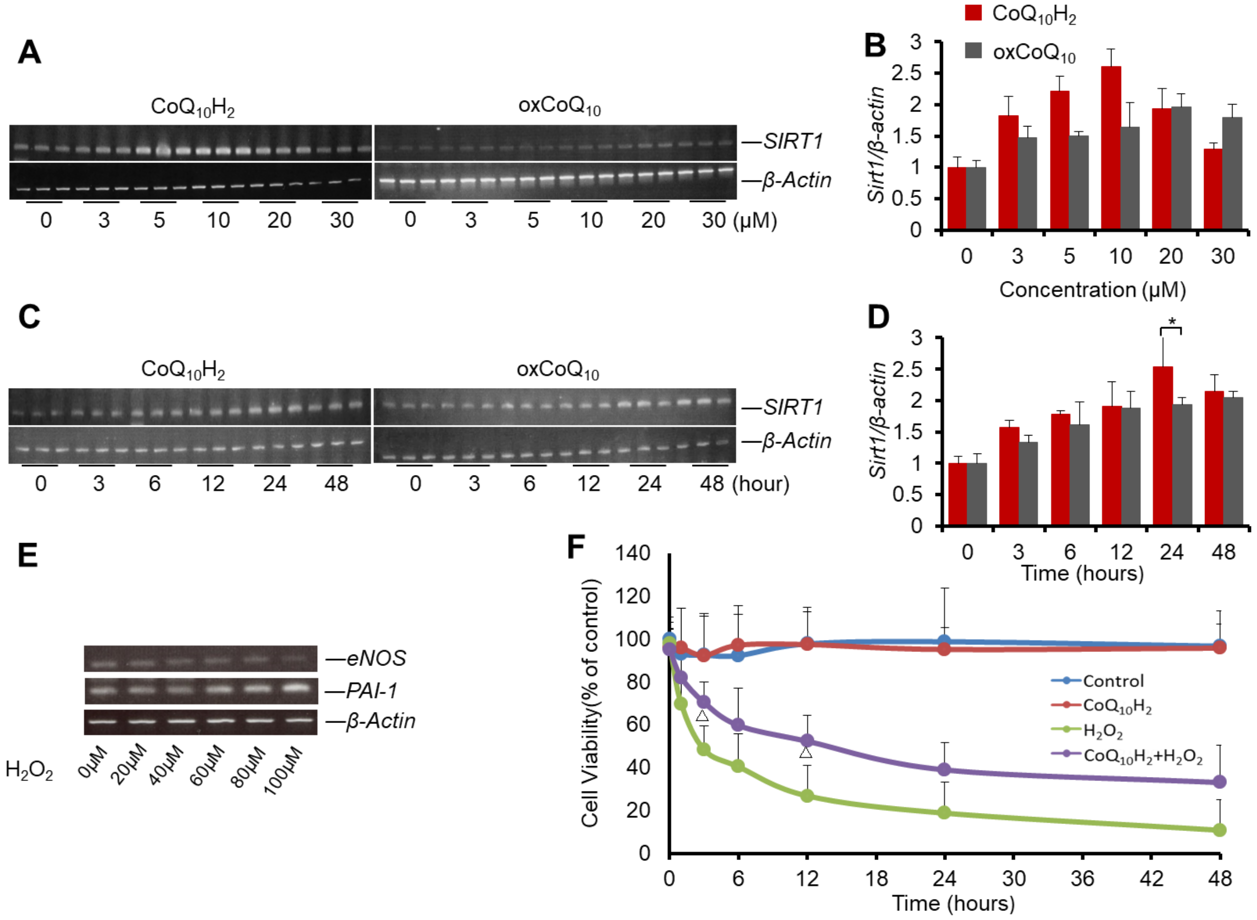

Figure S1. H_2O_2, $CoQ_{10}H_2$ and $oxCoQ_{10}$ affect HUVEC-related gene expression and $CoQ_{10}H_2$ prevents H_2O_2-mediated decreases in cell viability.

(A-D) HUVECs were treated with different concentrations of $CoQ_{10}H_2$ or $oxCoQ_{10}$ for different time periods. The expression level of *SIRT1* mRNA was measured by RT-PCR (n=3). *$p < 0.05$; mean ± SD, Student's t-test.

(E) *eNOS* and *PAI-1* expression in H_2O_2-treated HUVECs were determined by RT-PCR. **(F)** Cell viability in each group of HUVECs was determined by MTT (n=4). △$p < 0.05$ vs. H_2O_2 group; mean ± SD, one-way ANOVA followed by Tukey's test.

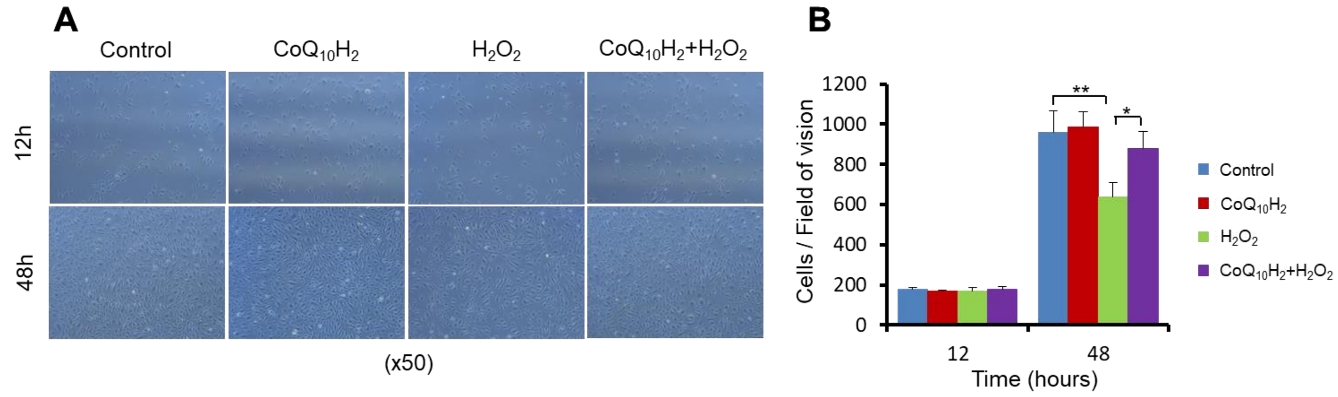

Figure S2. Preincubation with $CoQ_{10}H_2$ prevented H_2O_2-induced reduction of cell proliferation in HUVECs.

(A) Representative images of cells from each group (Control, $CoQ_{10}H_2$, H_2O_2 and $CoQ_{10}H_2 + H_2O_2$) at 12 or 48 hours. **(B)** Histograms show changes in the average number of cells in each group per field of vision after 12 and 48 hours (n=3). *$p < 0.05$, **$p < 0.01$; one-way ANOVA followed by Tukey's test.

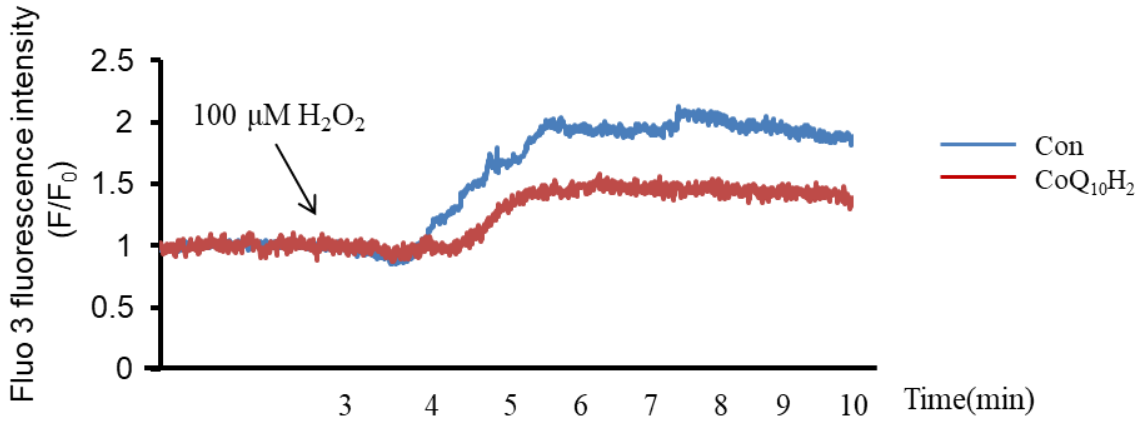

Figure S3. $CoQ_{10}H_2$ blocks H_2O_2-induced increases in free cytosolic Ca^{2+} levels in endothelial cells.

HUVECs were incubated for 24 hours with vehicle or 10 μM $CoQ_{10}H_2$ and treated with 100 μM H_2O_2. Ca^{2+} levels were determined by fluorescence microscopy using the probe Fluo-3-AM (n = 6).

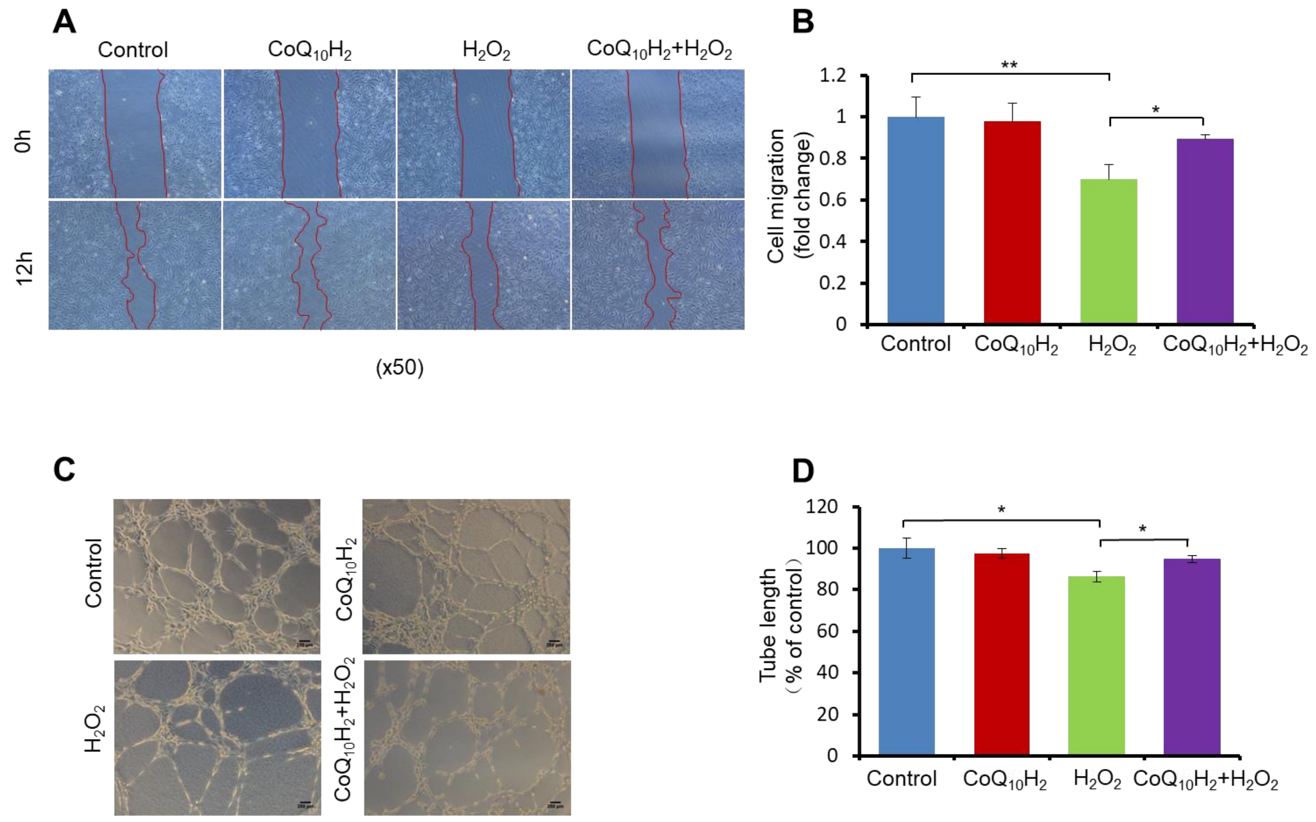

Figure S4. $CoQ_{10}H_2$ treatment prevented H_2O_2-induced reduction in migration and tube formation.

(A) Representative images of cell migration analysis evaluated using a wound healing assay conducted over 12 hours in 60 μM H_2O_2-induced reduction of migration. **(B)** Histograms show fold-change in migration activity relative to control cells (n=3). **(C)** Representative images from a 60 μM H_2O_2-induced tube formation assay after a 6 hours incubation. **(D)** Histograms show fold-change in total cell tube length relative to control cells (n=3). *$p < 0.05$, **$p < 0.01$; mean ± SD, one-way ANOVA followed by Tukey's test.

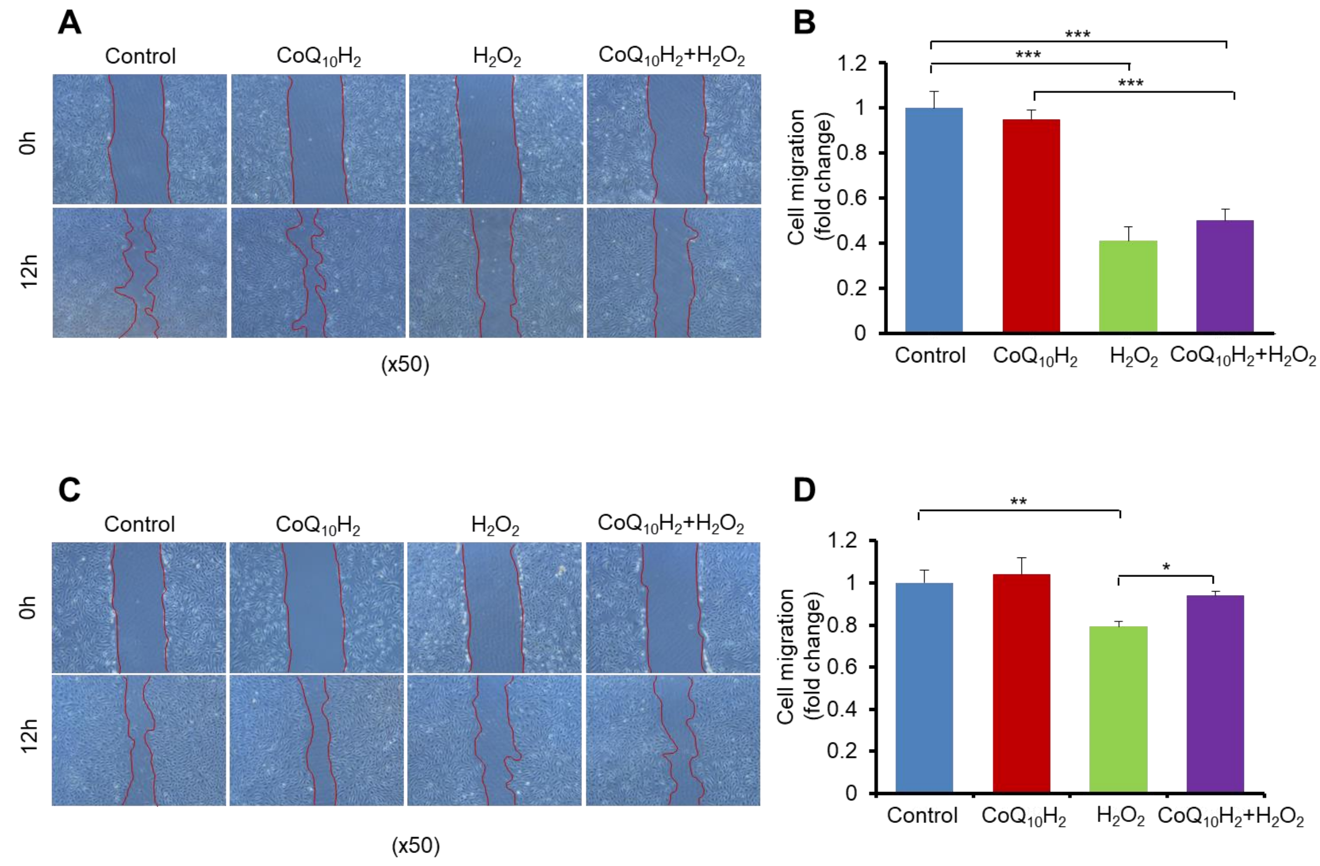

Figure S5. $CoQ_{10}H_2$ affects H_2O_2-dependent reduction in endothelial cell migration.

(A and **B)** Cultured HUVECs were pre-stimulated with 100 μM H_2O_2 for 12 hours or **(C** and **D)** 3 hours and then incubated with 10 μM $CoQ_{10}H_2$ for 24 hours. Cell migration was evaluated by a wound healing assay (n=3). *$p < 0.05$, **$p < 0.01$, ***$p < 0.001$; mean ± SD.

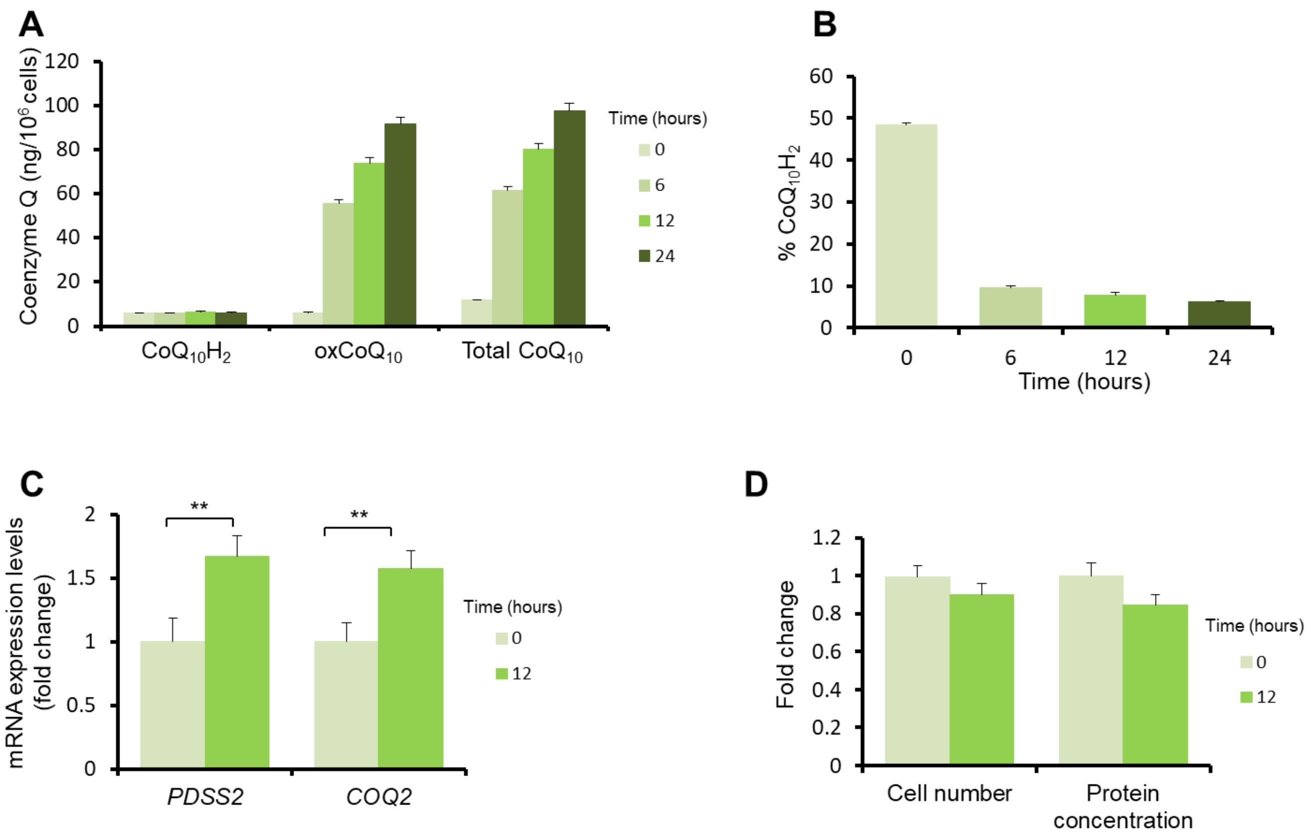

Figure S6. $CoQ_{10}H_2$ and $oxCoQ_{10}$ levels in HUVECs increased after H_2O_2 treatment.

(A) Time-dependent increases in $oxCoQ_{10}$ levels in HUVECs treated with H_2O_2. HUVECs were treated with 100 μM H_2O_2 for 0-24 hours. **(B)** The percentage of $CoQ_{10}H_2$ in total coenzyme Q10 (Total CoQ_{10}) was calculated (n=4). **(C)** Analysis of *PDSS2* and *COQ*$_2$ gene expression. Histograms show fold-change in mRNA level relative to control cells (n=3). $^{**}p < 0.01$; mean ± SD, Student's t-test. **(D)** Analysis of cell number and protein concentration. Histograms show fold-change in cell number and protein concentration relative to control cells (n=3).
